# Mitigation Strategies for Pandemic Influenza A: Balancing Conflicting Policy Objectives

**DOI:** 10.1371/journal.pcbi.1001076

**Published:** 2011-02-10

**Authors:** T. Déirdre Hollingsworth, Don Klinkenberg, Hans Heesterbeek, Roy M. Anderson

**Affiliations:** 1 MRC Centre for Outbreak Control and Analysis, Department of Infectious Disease Epidemiology, Imperial College London, London, United Kingdom; 2 Theoretical Epidemiology, Department of Farm Animal Health, Faculty of Veterinary Medicine, Utrecht University, Utrecht, The Netherlands; University of New South Wales, Australia

## Abstract

Mitigation of a severe influenza pandemic can be achieved using a range of interventions to reduce transmission. Interventions can reduce the impact of an outbreak and buy time until vaccines are developed, but they may have high social and economic costs. The non-linear effect on the epidemic dynamics means that suitable strategies crucially depend on the precise aim of the intervention. National pandemic influenza plans rarely contain clear statements of policy objectives or prioritization of potentially conflicting aims, such as minimizing mortality (depending on the severity of a pandemic) or peak prevalence or limiting the socio-economic burden of contact-reducing interventions. We use epidemiological models of influenza A to investigate how contact-reducing interventions and availability of antiviral drugs or pre-pandemic vaccines contribute to achieving particular policy objectives. Our analyses show that the ideal strategy depends on the aim of an intervention and that the achievement of one policy objective may preclude success with others, e.g., constraining peak demand for public health resources may lengthen the duration of the epidemic and hence its economic and social impact. Constraining total case numbers can be achieved by a range of strategies, whereas strategies which additionally constrain peak demand for services require a more sophisticated intervention. If, for example, there are multiple objectives which must be achieved prior to the availability of a pandemic vaccine (i.e., a time-limited intervention), our analysis shows that interventions should be implemented several weeks into the epidemic, not at the very start. This observation is shown to be robust across a range of constraints and for uncertainty in estimates of both *R_0_* and the timing of vaccine availability. These analyses highlight the need for more precise statements of policy objectives and their assumed consequences when planning and implementing strategies to mitigate the impact of an influenza pandemic.

## Author Summary

In the event of an influenza pandemic which has high mortality and the potential to spread rapidly, such as the 1918–19 pandemic, there are a number of non-pharmaceutical public health control options available to reduce transmission in the community and mitigate the effects of the pandemic. These include reducing social contacts by closing schools or postponing public events, and encouraging hand washing and the use of masks. These interventions will not only have a non-intuitive impact on the epidemic dynamics, but they will also have direct and indirect social and economic costs, which mean that governments will only want to use them for a limited amount of time. We use simulations to show that limited-time interventions that achieve one aim, e.g., contain the total number of cases below some maximum number of treatments available, are not the same as those that achieve another, e.g., minimize peak demand for health care services. If multiple aims are defined simultaneously, we often see that the optimal intervention need not commence immediately but can begin a few weeks into the epidemic. Our research demonstrates the importance of tailoring pandemic plans to defined policy targets with some flexibility to allow for uncertainty in the characteristics of the pandemic.

## Introduction

In the event of the emergence of a new human influenza A strain with a high case fatality rate indicating the possibility of a global pandemic with severe impact, control strategies primarily aim at limiting morbidity and mortality rather than halting transmission completely. This is because transmission of influenza A is difficult to block due to its short generation time and efficient transmission characteristics [1]. In the early days of the H1N1 influenza pandemic in Mexico in 2009 [2], social distancing measures were implemented with the aim of slowing the epidemic during its early stages. For any future pandemic of a directly-transmitted infectious agent, it is expected that similar strategies will be used in high resource settings while the pathogen is being identified, epidemiological studies to both characterize transmission [3], [4], [5], [6], [7] and determine pathogenicity are completed [8], [9] and strain-specific control options, such as vaccines, are being developed [10], [11]. For influenza, policy *options* are clearly outlined in national pandemic plans, but there is rarely any clear statement of policy *objectives* [12]. The problem is that these different objectives are potentially conflicting in their effects, and clear prioritisation is therefore necessary. Is the aim to minimize mortality and morbidity, is it to limit the peak prevalence of serious disease so that public health resources are not overwhelmed or is it to minimise the impact of the intervention on society and economy? In this paper we form a framework for policy makers to consider these potentially conflicting objectives.

A number of studies have investigated the role of targeted interventions at different phases of the epidemic based on mathematical models which include various levels of population structure and spatial complexity [13], [14], [15], [16], [17], [18], [19], [20]. However, none of these studies have addressed how multiple policy objectives are met by the common interventions, or how a clear statement of the key policy aims guides which set of interventions work best. It is typically assumed by policy makers that the more intervention measures implemented as early as possible in the course of the epidemic the better the outcome in terms of mitigation. Reservations about this strategic approach rest on the costs, and societal impact plus economic implications of sustaining control measures over a long period of time. In recognition of this the USA pandemic plan, for instance, mentions a maximum duration of 12 weeks for many transmission-reducing interventions [21]. However, there has been no quantitative analysis of when such an intervention should be initiated. Should it be as soon as the first cases are discovered, or later in the outbreak when more cases have arisen? Neither has it been acknowledged that planned levels of coverage with antiviral treatment or pre-pandemic vaccines may implicitly determine the magnitude of social distancing interventions required.

Studies have shown that during the 1918–19 influenza pandemic public health control strategies and changes in population contact rates lowered transmission rates and reduced mortality and case numbers [22], [23]. Similar measures were arguably effective for H1N1 in Mexico in 2009 [3]. Strategies used then, and to be considered in future, include social distancing measures, such as school closures [24], [25], restaurant and cinema closures [26], and transport restrictions [27], [28], [29]. There are a number of other measures, such as hand washing and the use of face masks [30], which may reduce contact rates [31], [32]. Transmission will also be affected by changes in human behaviour in response to a pandemic, as was observed in travel and mixing patterns during the severe acute respiratory syndrome (SARS) outbreak in 2003 [29].

**Figure 1:**
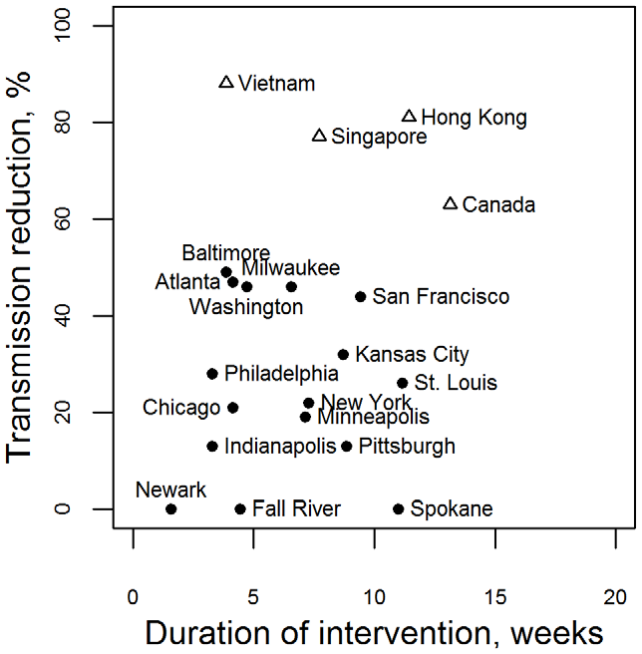
Magnitude and duration of responses to previous severe mortality outbreaks. Estimates of the reduction in the reproduction number and the duration of interventions during responses to the SARS outbreak in 2003 by country [18] (open triangles) and during the 1918 influenza pandemic in cities in the USA [22] (closed circles). A transmission reduction of 0% reflects an intervention which was estimated to have no effect on transmission.

Within the last 100 years, there have been two international outbreaks of a directly transmitted pathogen with high case fatality rates in which social distancing measures were implemented. The first was the influenza pandemic of 1918, where non-pharmaceutical public health strategies were effective at reducing morbidity and mortality in a number of settings [22]. However, the impact of these interventions on transmission was highly variable. An analysis of cities in the USA showed reductions in transmission ranged from approximately 0–60% (Figure 1). These interventions were held in place from 1 week to 3 months. One might expect that interventions with higher impact were held in place for shorter time, but there was no systematic relationship between the duration and the impact of interventions (Figure 1, black circles).

During the SARS outbreak of 2003, the aim of intervention strategies was to eliminate transmission, not only to mitigate the effects of the epidemic. Elimination was possible due to the characteristics of the virus - post-symptomatic transmission and a long generation time [1]. Large scale reductions in the transmission rate of SARS (>70%, Figure 1, [33]) were brought about by a number of public health interventions. These interventions were held in place for several weeks. The small amount of data available perhaps suggest a trend towards lower impact interventions being held in place for longer to achieve elimination (Figure 1, open triangles), but an important driver of the duration of these interventions was the number of cases that were present when the interventions started. These empirical data from two severe outbreaks suggest that moderate reductions in influenza transmission can be achieved and maintained at a population level for a number of weeks.

The impact of any particular intervention is difficult to estimate from past epidemics due to variation in the viral strain and its transmission properties, and due to the concurrent effects of many different behavioural responses and government led initiatives. Planning therefore depends increasingly on the predictions of mathematical models of viral spread that permit analyses of the potential impact of various interventions, alone or in combination [13], [34], [35], [36], [37], [38], [39].

In this paper we consider the effectiveness of contact-reducing interventions during the first six months after the initial cases, before a pandemic vaccine is available, and evaluate optimum interventions for a range of policy objectives or constraints, such as a limited stockpile of treatments or non-specific vaccine. Analyses are based on a mathematical model of virus transmission and the impact of control measures. We focus on the identification of policies that minimise peak demand for public health services and those which minimise the potential costs or socio-economic impact as evaluated by a simple cost function. This paper is not designed to give specific policy guidance. Box 1 outlines a number of factors which should be considered in designing policy which are not covered here. Our aim is to develop an understanding of how different policy objectives determine the optimal mix, timing of introduction and duration of implementation of the available mitigation strategies.

**Box 1.** Epidemic-Specific Characteristics Affecting Suitability of Interventions.

- Epidemiological characteristics of a future pandemic are not yet known and will be uncertain early in the epidemic. However, transmission estimates used for influenza pandemic planning proved to be close to those observed during the 2009 H1N1 pandemic [3]. Key parameters include *R_0_*, epidemic growth rate, generation time distribution, age-specific attack rate, asymptomatic case ratio, case fatality ratio, hospitalisation rates, treatment requirements, cross-immunity, drug resistance.
- Setting specific parameters will affect the growth rate and peak prevalence of an outbreak. These include age structure of the population, contact rates within and between age-groups, household structure, school attendance patterns, pre-existing immunity.
- Spatial structure may be important in certain settings, particularly population density, transport links and accessibility of health care services. Therefore interventions may be applied differently in different areas, depending on the spatial scale. Influenza growth rates are very rapid, so spread between areas could be rapid.
- The early course of an outbreak. When there are small numbers of cases and variable importation rates, there will be stochastic effects which will facilitate or slow the transition from localised outbreaks to exponential growth of the epidemic. This will affect the optimal timing of interventions.

## Methods

### Transmission model

All results have been obtained with a model based on the well-known deterministic SIR-model, that has proven its value in many studies of infectious diseases [40], [41]. We parameterized the model with a mean infectious period of 2.6 days (recovery rate γ = 1/2.6), and a basic reproduction number *R*_0_ = 1.8 (see Ferguson *et al* [13]) with a population of size *n* = 58.1 million. The population was subdivided into proportions of the population in the classes of *x* susceptibles, *y* infectives, and *z* immunes, with dynamics given by
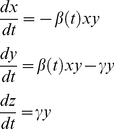
(1)The parameter 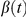 is the transmission rate, i.e. the number of contacts an infective has per day in which the infection is passed on, and has the baseline value 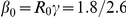. Simulations were started with 1 infective, *n*−1 susceptibles, and no immunes.

### Social distancing and epidemic dynamics

We investigated the impact of a social distancing intervention on transmission through a constant reduction in transmission, 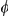, resulting from an unspecified combination of public health measures, maintained over a time period, *D*. In model terms, the transmission rate 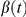 was assumed to change during intervention from the baseline rate 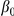 to a reduced rate 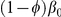. This happened from 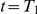, the start of the intervention, until 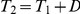, the end of the intervention of duration *D*. For the duration we considered three options, first an intervention that is kept in place indefinitely, second an intervention with a fixed duration of twelve weeks, which is the maximum duration mentioned in the USA national pandemic plan [21], and third an intervention until the a pandemic specific vaccine is available, after six months. In the ‘indefinite’ scenario, the duration of the epidemic was formally defined as the time until 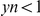.

Transmission-reducing public health interventions for influenza are unlikely to completely halt transmission [22], [23], [42], [43]. It is most likely that mitigation strategies will be ‘sub-critical’ interventions which reduce the effective reproduction ratio (the mean number of new infections per infected individual) towards, but not below, 1. Thus, we assumed that 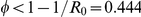. Numerical simulations of the model were used to evaluate the impact of the interventions twelve months after the first case. Impact is primarily measured by (the reduction in) the total number of cases. We also evaluated two other measures of effectiveness: firstly, the (reduction in) peak prevalence, since high prevalence may overwhelm public health facilities and as such increase both morbidity and mortality; and secondly, the socio-economic costs of the interventions, determined both by the level of intervention and the duration they are in place, calculated as the simple cost function 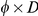.

### Antiviral drugs

Many countries have stockpiled antiviral drugs in preparation for an influenza pandemic [12]. Whilst these may be used prophylactically to reduce transmission [35], [44], [45], most pandemic strategies advocate the use of antivirals to treat cases of infection or to treat those cases where other risk factors suggest that disease severity may be high [46]. The treatment of cases will reduce morbidity and mortality and has been shown to be cost-effective for high risk patients [47]. We focus on the treatment of cases in combination with transmission-reducing intervention as above.

We make the assumption that treatment of cases does not affect transmission. The assumption is made firstly because drugs are given upon case notification, which is when much infectiousness may have passed [1], and secondly because symptomatic patients will be advised to remain at home reducing their contacts. The additional transmission reduction in transmission due to antivirals will thus be minor. The use of antivirals for severely ill patients could have implications for occupancy and therefore availability of isolation units and high dependency beds. Whilst this might change the infectious profile of the few severely ill patients who would have access to these facilities, it does not affect the majority of cases and detailed consideration of these logistics is outside the scope of this study. In addition, we do not include the possible effect of mass treatment on resistance [48] and therefore on the efficacy of the drugs. Consideration of these effects may lead to a range of different policy objectives, taking into account combination therapy or sequential deployment of different lines of therapy [49].

### Pre-pandemic vaccines

As well as stockpiling antivirals, it may be possible to reduce transmission and severity of disease by stockpiling a partially-protective pre-pandemic vaccine in advance of the pandemic [12]. Even partially effective vaccines can have large beneficial effects because the unvaccinated are indirectly protected from infection by those portions of the vaccinated population who are not infected or are less severely affected and possibly have reduced infectiousness (‘herd’ immunity – see [40]). Use of an imperfect vaccine can, however, also lead to increased incidence if reductions in infectiousness are associated with corresponding increases in the infectious period [50], [51]. Effectiveness estimates for a pre-pandemic vaccine are not available, but evidence from cross-protection studies led to the assumption that both susceptibility to infection and infectiousness may be reduced by 30% [13], [52]. The duration of infectiousness is assumed to be unchanged, precluding any increased incidence in the presence of the vaccine. We evaluate a partial vaccination strategy, in combination with a transmission reducing intervention, aiming to keep the number of unvaccinated cases (epidemic size) less than 25% of the population.

To consider vaccination with a pre-pandemic vaccine, the transmission model was adjusted to include infection of vaccinated individuals:
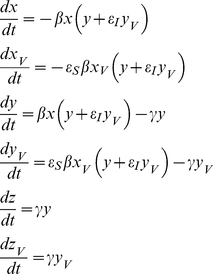
(2)In this adjusted model, 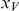, 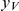, and 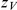 are the proportion of vaccinated individuals, and 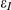 ( = 0.7) and 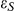 ( = 0.7) are the relative infectiousness and susceptibility of vaccinated versus unvaccinated individuals. It is assumed that vaccinated cases would not require treatment, and therefore were not included in the epidemic size or peak prevalence. Simulations were carried out with a vaccine coverage of 10%, starting with one unvaccinated infective.

### Scenarios

To place our results in a more realistic context whilst not giving precise policy guidance, we consider two scenarios for pandemic planning in high resource settings. They are scenarios which are covered in a number of pandemic plans. We will outline the range of interventions which can achieve these aims.

Scenario 1: A strain-specific vaccine is expected to be available within 6 months of the start of a pandemic. In order to minimize morbidity and mortality, social-distancing interventions will be used to ‘buy time’ until the vaccine is available. Antiviral drugs are available to treat symptomatic cases with a stockpile for up to 25% of the population. Social-distancing interventions will be used to ensure that symptomatic cases are kept below this level and to minimize socio-economic impact and peak demand for hospital and other public health services by minimizing prevalence in the population.

Scenario 2: This scenario is very similar to Scenario 1, except that in addition a pre-pandemic vaccine is available which can be rapidly rolled out to 10% of the population. The question of interest will be the extent to which the pre-pandemic vaccine will reduce the level of intervention required.

Since we are considering interventions implemented early in the epidemic, key epidemiological parameters may still be in the process of being estimated. Therefore, we investigated which strategies are least sensitive to incorrect estimation of *R*_0_, i.e. *R*_0_ = 1.7 or 2.0. In addition, availability of a pandemic vaccine may be delayed, or the pre-pandemic vaccine may be less effective than anticipated, so we ran our simulations out to an eight-month period and with a vaccine efficacy of 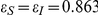 (50% less reduction in transmission).

## Results

We first investigate the impact of social distancing interventions alone. The received wisdom of outbreak control strategies is that the maximum level of control measures should be put in place as rapidly as possible. However, there may be delays before control strategies are implemented due to difficulties in identifying the early stages of a novel outbreak, as well as other logistical, political and economic constraints. Because the interventions considered here are sub-optimal, cases will continue to occur whilst the intervention is in place, but at a slower rate than in the unconstrained epidemic. This means controls may need to be held in place for a long time, which may be costly. Detailed derivations of the analytical results are given in a Text S1.

### Long-term interventions

**Figure 2:**
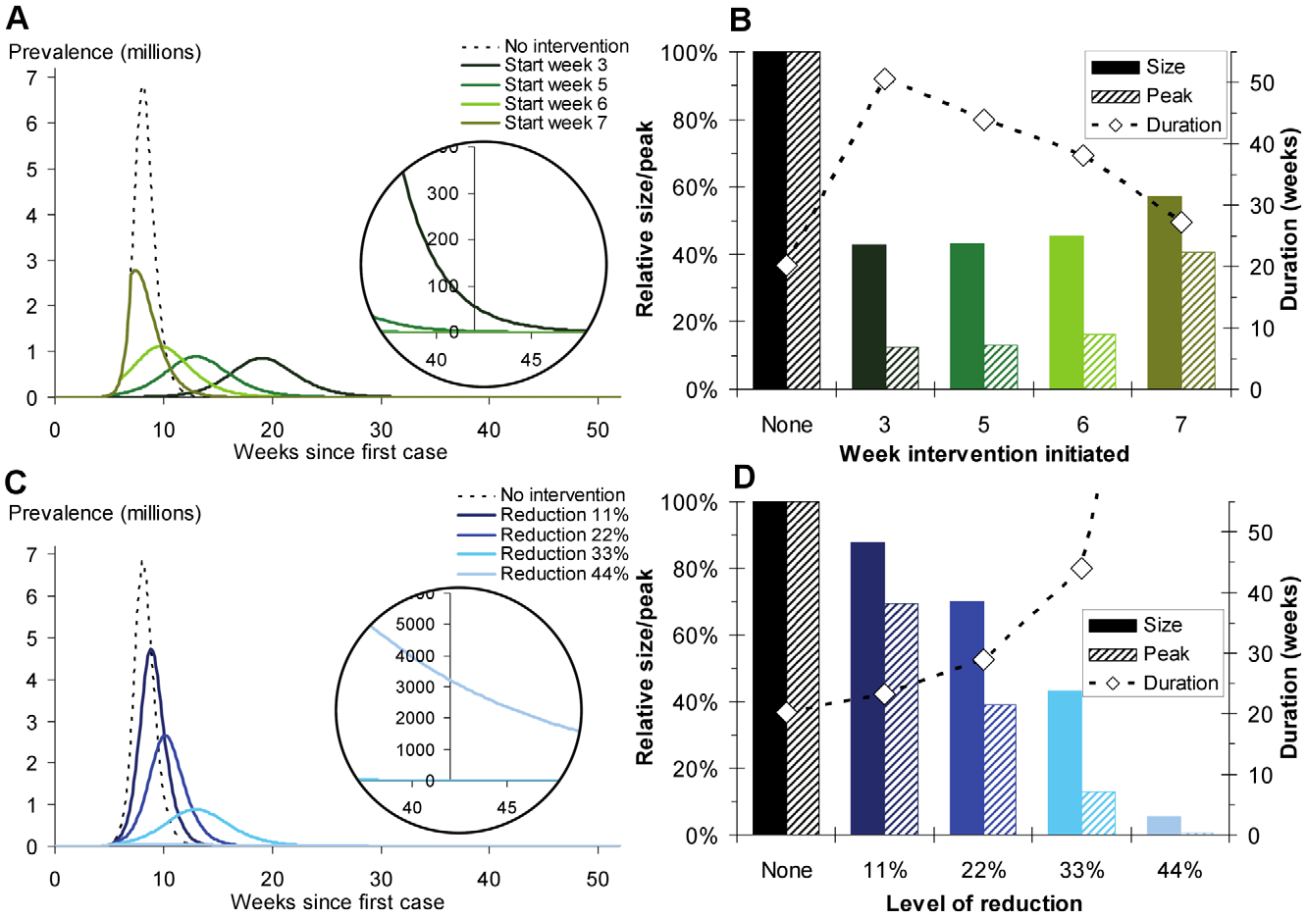
Effect of timing and strength of interventions on outcome for long-term interventions. **A**, **C**: Prevalence of infectious cases under different control scenarios (dotted line indicates unconstrained epidemic). **B**, **D** Effect of interventions on total number of cases (solid bars), peak prevalence (striped bars), and time until final case recovers (diamonds). **A**, **B** intervention commences week 3, 5, 6 and 7 with a 33.3% reduction in transmission. **C**, **D** intervention commences week 5 with 11.1%, 22.2%, 33.3% and 44.4% reduction in transmission.

One possible policy choice is to maintain an intervention irrespective of cost until the last case has recovered from the disease. This will always reduce the total number of cases and peak prevalence. These quantities can be expressed or approximated by analytical expressions, which we derive in Text S1 and illustrate using numerical simulations. The final proportion of the population affected by an unconstrained epidemic, *a_NI_*, is given by solving [40], [41]

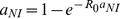
(3)The final size increases monotonically with increasing *R_0_* and does not depend on the generation time of the infection [40]. For a long term intervention, implemented at *T_1_* and held in place until there are no cases (Figure 2), the final epidemic size, 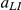 (proportion of the population who have been infected) is given by
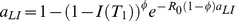
(4)where 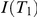 is cumulative incidence up to time *T_1_*. In the exponential growth phase, the cumulative incidence can be approximated by
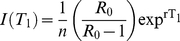
(5)where *r* is the epidemic growth rate, given by 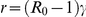.

For our parameter values, this approximation works well until about *T*_1_ = 49 days (7 weeks), when equation (5) overestimates 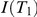 by 22%. The final epidemic size decreases monotonically as the timing of the intervention, *T_1_*, becomes earlier, and as the size of the intervention, 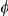, becomes larger (Figure 2). However, before week 5 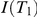 is very small, so interventions starting earlier do not have much effect (Figure 2).

In the absence of an intervention the maximum prevalence occurs when 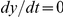, or when 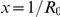, and the maximum prevalence is (using the equations above and 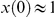 approximations to the initial conditions) is [40]

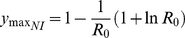
(6)which increases with increasing *R_0_*, and, as with the unconstrained epidemic size, does not depend on the generation time.

In the presence of the intervention, maximum prevalence is dependent on the proportion of the population who are still susceptible at the time of the intervention. If the intervention is initiated before the peak in the unconstrained epidemic, and if cumulative incidence is sufficiently high and the proportion of the population still susceptible at the start of the intervention is less than 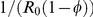, then peak prevalence will be at the start of the intervention, 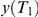.

On the other hand, if the cumulative incidence is less than 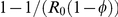 there will be a peak during the intervention (Figure 2), which is given by
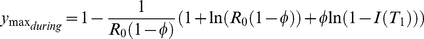
(7)If the intervention is initiated after the peak of the unconstrained epidemic, then there will not be another peak in prevalence during the intervention, since there will be too few susceptible individuals.

These analytical results can be used to understand the effect of an intervention on the final size and peak prevalence, but we do not have neat expressions for the resulting duration of the whole epidemic (time until final case recovers) when an intervention is in place, and therefore we turn to simulation (Figure 2). The higher the transmission rate, the shorter the epidemic, which may be a desirable policy outcome.

For influenza-like parameters, a few weeks delay may have only moderate deleterious consequences for peak prevalence, peak incidence or epidemic size (Figure 2). This delay will result in higher peak prevalence, but it will also result in a considerably shorter epidemic than an early intervention (Figure 2A circular inset and 2B). This may be a desirable outcome in economic terms. The level of reduction in transmission has similar effects, where a more effective intervention put in place early in the epidemic will lead to the smallest epidemic size and peak prevalence, but the longest epidemic duration (Figure 2C and D).

In brief, the earlier a long term intervention is put in place and the more effective it is at reducing transmission, the greater the beneficial effect in terms of total epidemic size and peak prevalence. Interventions of this kind are likely to be the most costly, and, counter-intuitively, may have to be held in place the longest. A strong argument to start an intervention early, however, is that the epidemic peak occurs later for early interventions (Figure 2A), allowing time to prepare public health facilities, to manufacture a strain specific vaccine and because there is great uncertainty about severity in the early stages of an outbreak [8].

### Short-term interventions

The drawbacks of a long intervention period are recognised in the USA national pandemic plan, where a maximum duration of 12 weeks intervention is anticipated - another policy choice we considered. As above, we first consider some analytical expressions, and illustrate them using numerical simulation.

**Figure 3:**
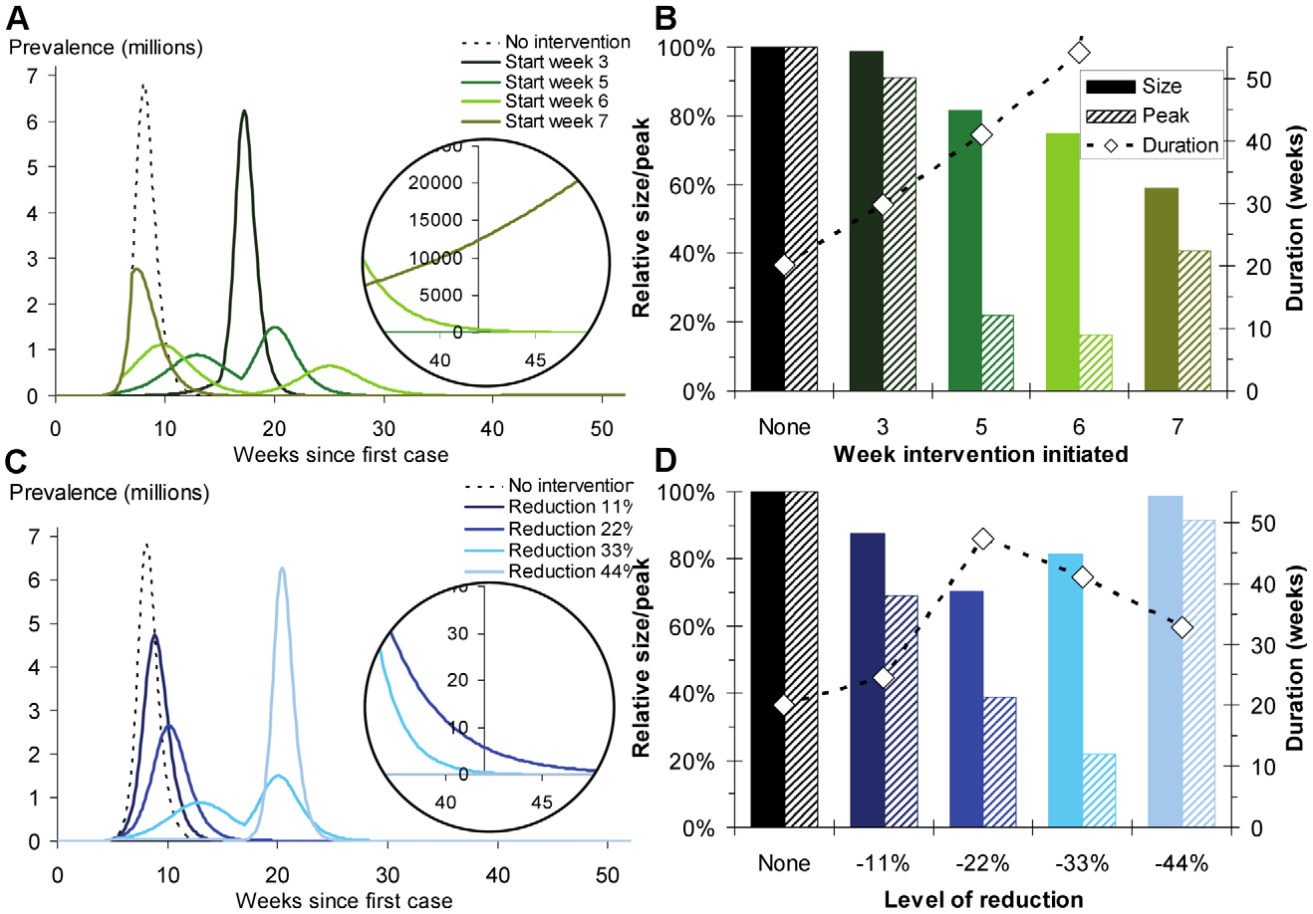
Effect of timing and strength of interventions on outcome for an intervention of 12 weeks. **A**, **C**: Prevalence of infectious cases under different control scenarios with dotted line indicating unconstrained epidemic. **B**, **D** Effect of interventions on total number of cases (solid bars), peak prevalence (striped bars), and time until final case recovers (diamonds). **A**, **B** intervention commences week 3, 5, 6 and 7 with a 33.3% reduction in transmission. **C**, **D** intervention commences week 5 with 11.1%, 22.2%, 33.3% and 44.4% reduction in transmission.

For a single short term intervention from 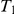 to 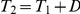, the final epidemic size, 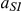, is given by
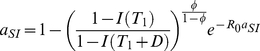
(8)Note that, although 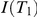 can still be approximated during the exponential phase of the epidemic (equation (5)), we cannot approximate 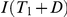. In this case, the relationship between the final epidemic size and intervention parameters is more complex because cumulative incidence at the time the intervention is lifted depends both on cumulative incidence at the time the intervention is initiated and the size of the intervention, 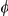. For example, if the duration of the intervention and its starting time are fixed, the epidemic size is optimized for intermediate values of the size of the intervention, 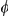 (Figure 3B, D).

With a short-term intervention, there are three possible maximum prevalence points. Firstly, prior to the intervention (equation (6)), during the intervention (equation (7)), or after the intervention
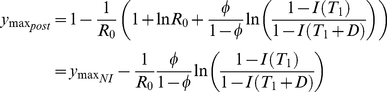
(9)(note that 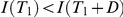). The peak value could also occur at the point at which the intervention starts, i.e. when 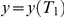. The conditions for each peak being the maximum are given in Table 1. A large magnitude intervention (large 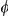) may actually be deleterious, leading to a larger resurgence in prevalence after the intervention than an intervention with a smaller reduction in transmission.

**Table 1:** Maximum prevalence in the presence of an intervention.

Condition	Maximum prevalence	Local peak prior	Increasing prevalence during	Local peak during	Local peak post
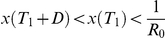	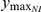	Y	N	N	N
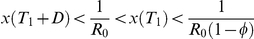	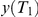	N	N	N	N
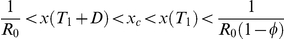	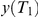	N	N	N	Y
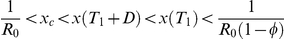	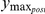	N	N	N	Y
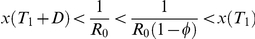	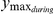	N	Y	Y	N
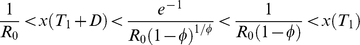	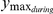	N	Y	Y	Y
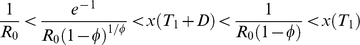	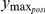	N	Y	Y	Y
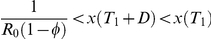	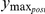	N	Y	N	Y

The interventions range from late interventions at the top of the table to short and early interventions at the bottom of the table. Note that 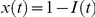 and 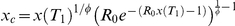.

With a short-term intervention, there is no longer a monotonic relationship between the policy outcomes and the magnitude and length of the intervention. Therefore strategies which contain the epidemic size below certain levels are unlikely to be the same interventions which contain peak prevalence below particular targets.

For influenza-like parameters a 12-week intervention will almost certainly lead to a resurgence of the epidemic once the controls are lifted (Figure 3A, C). If peak prevalence is very much lower during the intervention than it would be with no intervention, the implemented policy may even result in almost no change in the total epidemic size (Figure 3). For late, or less effective, interventions, prevalence during the intervention is higher than for early, or more effective, interventions,, resulting in fewer susceptible individuals remaining when the intervention is lifted. In this case the second peak is smaller, and reductions in total epidemic size are larger (Figure 3).

For short term interventions, in contrast to long-term strategies, peak prevalence, peak incidence, and epidemic size cannot all be minimized by the same strategy. For instance, a 33% reduction in transmission timed to minimise total epidemic size (Figure 3A, B, initiated week 7) may not be the intervention which minimises peak prevalence (Figure 3A, B, initiated week 6). Both these strategies have small and late resurgent epidemics (Figure 3A, circular inset), with cases beyond the end of the year. Similarly, an intervention initiated at week 5 may minimise peak prevalence for a 33% reduction in transmission (Figure 3C, D), or minimize epidemic size with a 22% reduction in transmission (Figure 3C, D), but neither of these strategies are optimal if the aim is to have the epidemic exhaust itself most rapidly, with the quickest epidemic being the one without any intervention.

The intervention always reduces peak prevalence from what it would have been in the absence of an intervention. However, which particular value is the peak value is determined by the timing of the intervention and the magnitude of the intervention (Table 1). Each of these vary according to the characteristics of the intervention, and the underlying epidemic. For a fixed starting time and duration, there is a non-linear relationship between peak prevalence and the reduction in transmission, 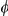 (Figure 3). The value of 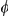 for which peak prevalence is minimized is almost certainly not that at which the total epidemic size is minimized (Figure 3).

### Scenario 1: Limited antivirals, 6 months to vaccine availability

It is not possible to achieve a symptomatic epidemic size of 25% of the population with a 12 week intervention for these parameter values. We therefore consider a scenario in which an intervention is initiated in the first weeks or months of the outbreak and held in place until 6 months after the start of the outbreak.

**Figure 4:**
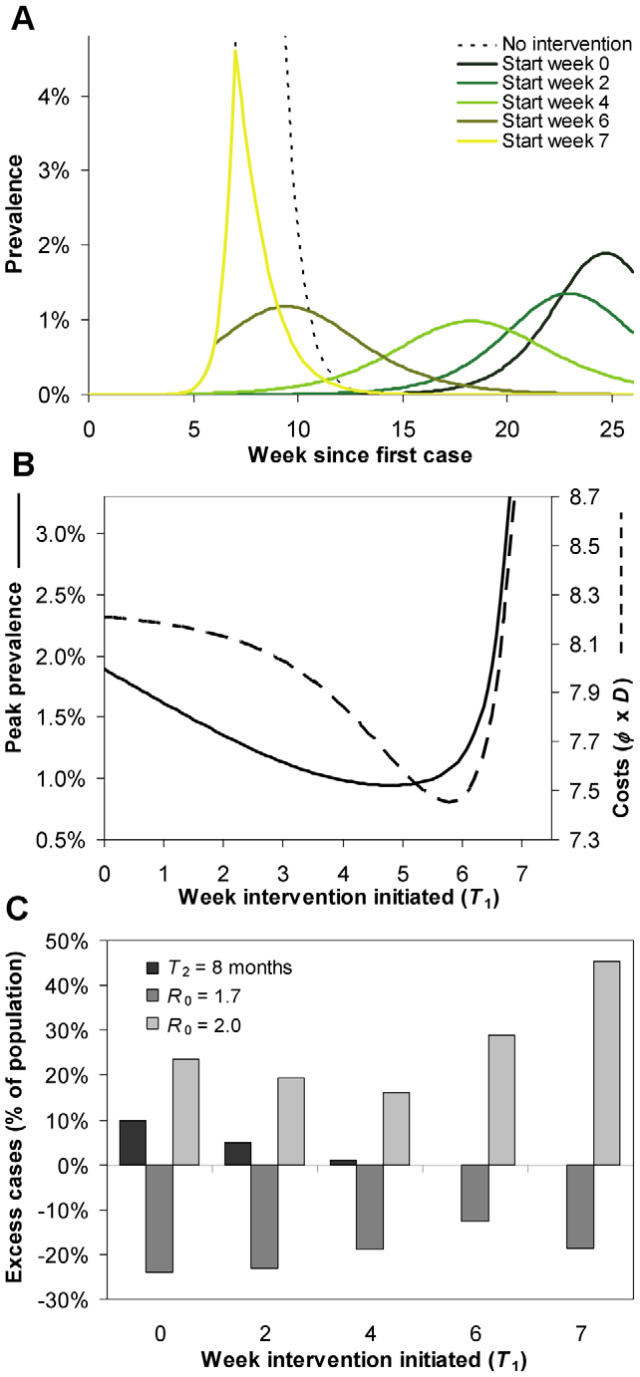
Comparison of intervention strategies which ‘buy time’ until a strain-specific vaccine is available 6 months into the epidemic and contain symptomatic cases to utilize a stockpile of treatments for 25% of the population. **A** Uncontrolled epidemic (black dotted curve) and epidemic curves for five different strategies, starting at different times: *T*_1_ = 0, 2, 4, 6, or 7 weeks into the epidemic. The required reductions in transmission are 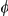 = 32%, 34%, 36%, 37% and 49%. **B** Peak prevalence (solid curve) and costs of interventions calculated as 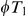 (dashed curve), in relation to the time of commencement of intervention. **C** Excess number of cases for the five strategies if the parameters of the epidemic are different to those for which these interventions were designed: the availability of a strain specific vaccine is delayed until 8 months (black), transmission has been overestimated and *R*_0_ = 1.7 (dark grey), or transmission has been underestimated and *R*_0_ = 2 (light grey).

Many different interventions can be used to constrain the epidemic size to 25% of the population. They range from an early intervention with a mild reduction in transmission, to a late, more impactful intervention (Figure 4A). To achieve this aim whilst minimising peak prevalence it is not necessary to initiate the intervention early, in fact a delay may even be beneficial (Figure 4B). But, the intervention must start before 7 weeks (for these parameter values), when the number of cases prior to the intervention becomes large.

If we evaluate the socio-economic ‘cost’ of these interventions as a simple product of the duration of the intervention and the reduction in transmission achieved, a delay also reduces the costs of the intervention, and the ideal intervention is more clearly defined (Figure 4B). Delay is valuable because transmission is being reduced, not eliminated, and therefore some of the effort in constraining the epidemic at the early stages is redundant.

Choices about intervention policy will be made early in the epidemic when parameters are uncertain. For example, *R_0_* and the date of availability of the vaccine could be over or under estimated. Of course, designing this intervention based on an overestimate of R0 means that the epidemic is smaller than expected, and so the intervention is too large and there are fewer cases overall (Figure 4C). An underestimate in *R_0_* means that the epidemic is larger than expected and so the intervention is not large enough to contain the epidemic and there are more cases than expected (Figure 4C). In either of these cases, the intervention would have to be adjusted during the outbreak. If the ‘optimum’ intervention, which minimised peak prevalence, is chosen, it is more robust to changes in R0 than the other options (Figure 4C). A delay in the availability of vaccine increases the number of cases, but picking a late intervention minimises this effect.

### Scenario 2: Additional benefit of pre-pandemic vaccine

**Figure 5:**
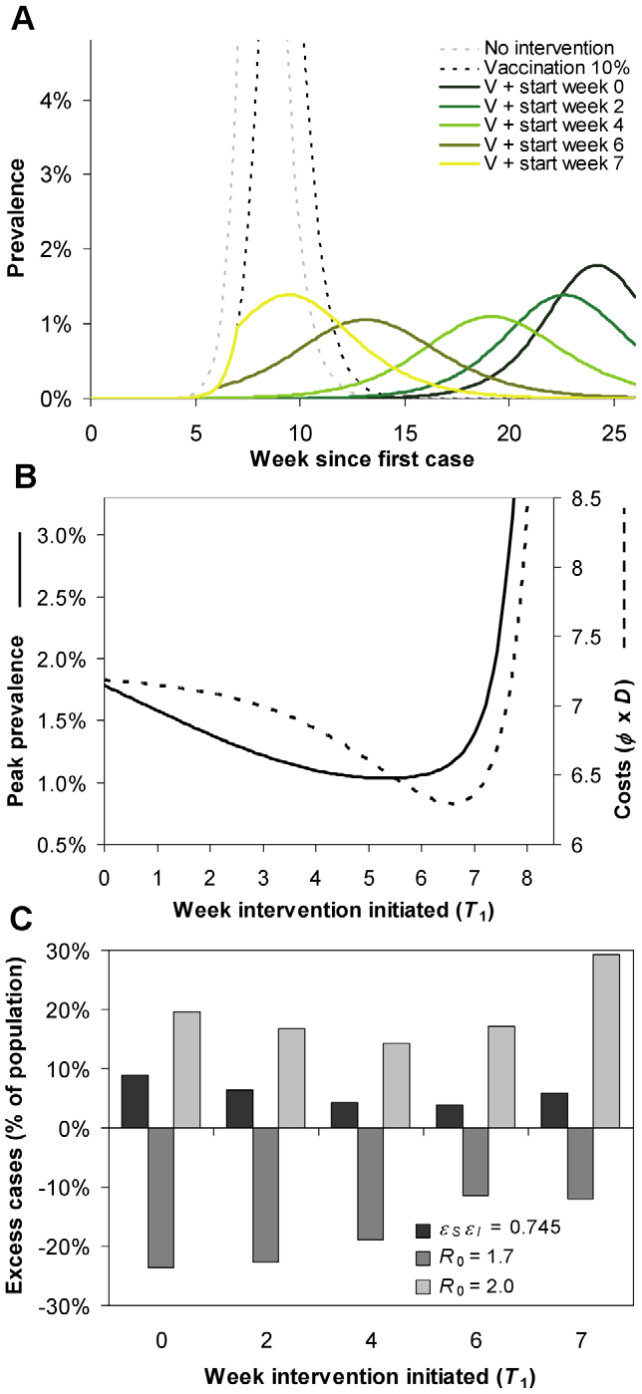
Addition of a pre-pandemic vaccine for 10% of the population. Comparison of intervention strategies which ‘buy time’ until a strain-specific vaccine is available 6 months into the epidemic and contain symptomatic cases to utilize a stockpile of treatments for 25% of the population when 10% of the population are vaccinated with a vaccine which reduces susceptibility and infectiousness by30%. **A** Uncontrolled epidemic (black dotted curve) and epidemic curves for five different strategies, starting at different times: *T*_1_ = 0, 2, 4, 6, or 7 weeks into the epidemic. The required reductions in transmission are 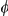 = 28%, 30%, 31%, 32% and 33%. **B** Peak prevalence (solid curve) and costs of interventions calculated as 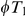 (dashed curve), in relation to the time of commencement of intervention. **C** Excess number of cases for the five strategies if the parameters of the epidemic are different to those for which these interventions were designed: the pre-pandemic vaccine is less effective (black), transmission has been overestimated and *R*_0_ = 1.7 (dark grey), or transmission has been underestimated and *R*_0_ = 2 (light grey).

Use of an imperfect vaccine for only 10% of the population results in a slower epidemic with fewer cases (Figure 5). The use of a pre-pandemic vaccine means that interventions which contain the total number of cases and peak prevalence can be rolled out later (Figure 5A), compared to the non-vaccination scenario. Also, as can be seen from the simple cost function (Figure 5B), the level of intervention can be reduced if pre-pandemic vaccines are used. The true economic value of this reduction in costs depends on the relative costs of vaccination, cases and interventions. The general picture remains the same as without vaccination. To minimize peak prevalence, the intervention should be initiated earlier than to minimize costs, but both objectives require interventions that commence several weeks into the epidemic growth phase (Figure 5B). Sensitivity to the value of *R*_0_ or the effectiveness of the pre-pandemic vaccine highlights that once again the most robust strategies are those that are minimize peak prevalence (Figure 5C).

## Discussion

In the absence of detailed analyses, it is often argued that epidemic outbreak control is best achieved by putting all mitigation options into play as early as is feasible. There may be delays before control strategies are implemented due to difficulties in identifying the early stages of a novel outbreak [53], as well as other logistical, political and economic constraints. Of course, if interventions are held in place until a pandemic vaccine is available a greater level of reduction and earlier start of intervention will result in fewer cases, and a lower peak prevalence and incidence if intervention starts before the peak. However, not only are the costs of an intervention held for a long time likely to be high, but high demand for health services will be extended over a longer time period. Our results indicate that an intervention starting at a few weeks into the epidemic is almost as effective at reducing epidemic size and peak prevalence as one starting at week 0. As such, given that the social and economic burden will be greater when starting earlier, starting a little bit later may be a better policy option. However, this will crucially depend on the socio-economic costs of both cases and interventions and on the estimated severity of the epidemic, which may be uncertain in the early stages of the epidemic [8].

As noted in the introduction, the drawbacks of a long intervention period are recognised in the USA national pandemic plan, where a maximum intervention duration of twelve weeks is anticipated [21]. Using a twelve-week intervention, we have illustrated how the introduction of a short term intervention complicates the dynamics and increases the potential for conflict between policy aims. Interventions of limited duration are very likely to result in a resurgence of the epidemic once they are lifted, unless it is imposed late in the epidemic or with low effectiveness. However, the height of this resurgence can be managed. A twelve-week interventions minimizing peak logistical pressure (peak prevalence and incidence) need not be very strong but require a timely start. On the other hand, an intervention that minimizes total epidemic size needs to be stronger and can start later, preventing a second peak.

A number of American cities experience a second peak in mortality following the lifting of interventions during the 1918 pandemic [22], [43]. Re-analyses of a number of cities showed that multiple interventions were more effective at controlling transmission than single interventions [43]. In addition, it was found that the later multiple interventions were implemented, the less effective they were in reducing mortality [22], [43]. This was most notable when controls were implemented when excess mortality was higher than ∼100 per 100,000 [22]. This conclusion cannot be so easily drawn in epidemics for which interventions were initiated prior to this threshold [22]. Here, we have shown that for short term interventions implemented during this early part of the epidemic earlier commencement is not always better, and that the outcome is highly sensitive to the timing and effectiveness of interventions.

Our two scenarios for policy design illustrate that applying one objective and then another sequentially (e.g. limiting total cases and then minimising peak prevalence for that epidemic size) can be used to resolve potentially conflicting aims. Our results also show that the most extreme and earliest mitigation interventions are not always the best, and not always the least costly. It has not previously been highlighted that the level of stockpiles will quantitatively affect the required magnitude of social-distancing interventions so that all those who require treatment will receive it. Any level of stockpiled antiviral drugs will reduce morbidity and mortality and therefore reduces the need for transmission-reducing interventions, as not all cases need to be prevented, but the availability of drugs means that demand for these drugs should not exceed supply. In addition, our results illustrate that even low coverage with imperfect vaccines can lead to reductions in the required interventions level to meet a defined objective for control.

There are many complexities involved in quantifying the effect of interventions which are not included here, the complexities of transmission by age and spatial heterogeneities, the likely behavioral changes during an epidemic that affect transmission, seasonal variation in transmission, the logistics of delivery of pre-pandemic vaccines and drugs, the economic costs of an outbreak and potential development of resistance to antiviral drugs. Detailed investigations are required to tailor general policies to particular settings, and therefore we are not attempting to make quantitative policy recommendations (see Box 1). However, uncertainties with regard to characteristics of the next pandemic strain will make it difficult in general to do very detailed optimization analyses. Decisions on stockpiling must be based on knowledge from previous pandemics and seasonal influenza, but when a pandemic is at hand one has to work with the stockpiles available. Intervention measures can be additionally imposed if a shortage of drugs is expected, or lifted to reduce the impact of intervention on society and economy, if drug supplies permit. Our analyses show that there is indeed some time to choose the appropriate level of control, as very early commencement of intervention is hardly ever optimal for these time-limited interventions.

Our analyses also illustrates that even a simple inclusion of ‘costs’ changes what is optimal by comparison with analyses that are just based on impact on epidemiological measures. Economic costs typically enter the equations in a non-linear term as indicated in our model formulation. However, including empirically derived cost functions will probably lead to the inclusion of more highly non-linear functions. This highlights the need to include more robust economic constraints into future epidemiological model analyses for public health policy support. In our view, this is a more urgent need than that of increasing the complexity of epidemiological description within models of infectious disease control. Concomitantly, there is the associated need for measurement of the appropriate cost functions. Data is available for both drug and vaccine purchase but this is regarded as confidential at present as neither the pharmaceutical industry nor government health departments are keen to say how much was paid per dose as a function of total volume purchased. Future research must address the detail of cost and benefit, both in terms of measurement of direct and indirect socio-economic costs, the costs of stockpiling and the benefits of reducing the impact of the epidemic and in terms of using a template for analysis that reflects the dynamics of virus transmission and the impact of control measures.

In our model we have considered contact-reducing interventions, the use of antiviral medication, and vaccination with a pre-pandemic vaccine. For insight into the effect of other control options, it is useful to understand what characterizes these three particular control measures. Antivirals work on the individual level, contact reduction on the population level, and vaccination on both. Contact reduction and vaccination are preventive measures, whereas treatment is reactive. Treatment and vaccines require stockpiling, and both are flexible with respect to possible timings of introduction during the epidemic. Contact reduction is flexible in both planning and timing, but has major implications for the normal functioning of society.

This flexibility implies that a broad range of more complex strategies could be envisaged, for example implementing and lifting a hierarchy of controls in response to the dynamics of the epidemic and importation of cases. However, the simple scenarios illustrated here highlight the complexities in selecting the best intervention policy, in terms of magnitude, timing and duration of interventions. The optimum intervention in terms of minimising peak logistical pressures (peak prevalence or incidence), may not be the same as one which minimises total epidemic size, and will almost certainly not be the one minimising direct social or economic impact from the intervention itself. The aims of a public health intervention policy must therefore be clearly defined, so that in the early phase of a pandemic sufficient resources can be put into characterizing the virus strain and measuring key epidemiological parameters as an essential template for decisions on what is the optimal mitigation strategy.

## Supporting Information

Text S1 Detailed derivations of the mathematical expressions for epidemic size and peak prevalence.
(0.90 MB PDF)
