# Supplementary material for: Mitigation Strategies for Pandemic Influenza A: Balancing Conflicting Policy Objectives
Source: PLoS Comput Biol. 2011 Feb 10;7(2):e1001076. doi: 10.1371/journal.pcbi.1001076 (PMC3037387; doi:10.1371/journal.pcbi.1001076)
Supplement: Text S1 — Detailed derivations of the mathematical expressions for epidemic size and peak prevalence. (0.90 MB PDF) [file pcbi.1001076.s001.pdf]

Mitigation strategies for pandemic influenza A: balancing conflicting policy objectives

T. Déirdre Hollingsworth[1*], Don Klinkenberg[2*], Hans Heesterbeek[2], Roy M. Anderson[1]

[1]MRC Centre for Outbreak Control and Analysis, Department of Infectious Disease Epidemiology, Imperial College London, St Mary's Campus, London W2 1PG, UK. [2]Theoretical Epidemiology, Department of Farm Animal Health, Faculty of Veterinary Medicine, Utrecht University, Utrecht, The Netherlands.

[*] DH and DK contributed equally to this work

Text S1

This supplementary text contains derivations of the expressions contained in the results section in the main text. Equation numbers with the format (S.1) refer to equations in this document, other equation numbers refer to those in the main text. The results for the case without interventions have been derived in a number of textbooks (e.g. [40,41]).

1 No intervention

When no intervention is in place, expressions for the epidemic size and peak prevalence can be derived. The first step is to express *y* as a function of *x*. This is done by deriving an expression for dy/dx by dividing the equation for dy/dt by the equation for dx/dt (see equation (1)), giving

$$\begin{aligned}\frac{dy}{dx} &= \frac{\beta(t)xy-\gamma y}{-\beta(t)xy} = \frac{\gamma}{\beta(t)x}-1\\ &= \frac{1}{R_0 x}-1\end{aligned} \qquad \text{(S.1)}$$

when there is no intervention in place. And therefore, *y* may be expressed as function of *x* by integrating by parts,

$$\begin{aligned}\frac{dy}{dx} &= \frac{1}{R_0 x}-1\\ \int dy &= \int \frac{1}{R_0 x}-1dx\\ y &= \frac{1}{R_0}\ln x - x + c_1\end{aligned} \qquad \text{(S.2)}$$

where c_1 is a constant determined by the initial conditions. So, before interventions, or in the absence of interventions, the relevant initial condition is $y(0)\approx 0, x(0)\approx 1$ and so

$$\begin{aligned}0 &= \frac{1}{R_0}.0-1+c_1\\ c_1 &= 1\end{aligned} \qquad \text{(S.3)}$$

And so

$$y = 1 - x + \frac{1}{R_0}\ln x \tag{S.4}$$

1.1 Epidemic size

At the end of the epidemic with no intervention, as $t \to \infty$, there are no cases left, $y(\infty) \to 0$, and the total epidemic size, $a_{NI} = 1 - x(\infty)$, and therefore

$$\begin{aligned} 0 &= 1 - \left(1 + a_{NI}\right) + \frac{1}{R_0}\ln\left(1 - a_{NI}\right) \\ a_{NI} &= 1 - \mathrm{e}^{R_0 a_{NI}} \end{aligned} \tag{S.5}$$

1.2 Peak prevalence

Peak prevalence is calculated by considering where $dy/dx = 0$, from equation (S.1),

$$\begin{aligned} 0 &= \frac{1}{R_0 x} - 1 \\ x &= \frac{1}{R_0} \end{aligned} \tag{S.6}$$

The value of peak prevalence at this time is, from equation (S.4)

$$\begin{aligned} y_{\max_{NI}} &= 1 - \frac{1}{R_0} + \frac{1}{R_0}\ln\frac{1}{R_0} \\ &= 1 - \frac{1}{R_0}\left(1 + \ln R_0\right) \end{aligned} \tag{S.7}$$

Which is given as equation (6) in the main text.

1.3 Cumulative incidence

Cumulative incidence in the exponential phase of the epidemic may be calculated by approximating $x \approx 1$ in the equation for dy/dt (equation (1)). If the transmission rate is constant, the prevalence $y(t)$ is

$$\begin{aligned} &\frac{dy}{dt} = \beta y - \gamma y \\ &\Rightarrow y(t) = y(0)\exp\left(\left(\beta - \gamma\right)t\right) = \frac{1}{n}\exp\left(\left(R_0 - 1\right)\gamma t\right) \end{aligned} \tag{S.8}$$

As incidence is equal to βxy, cumulative incidence, $I(t)$, is approximated by

$$\begin{aligned}&\frac{d}{dt}I=\beta xy\approx\beta\frac{1}{n}\exp\left(\left(R_0-1\right)\gamma t\right)\\&\Rightarrow I\left(t\right)=\frac{1}{n}\frac{R_0}{\left(R_0-1\right)}\exp\left(\left(R_0-1\right)\gamma t\right)\end{aligned}\qquad\text{(S.9)}$$

which is used to derive equation (5) in the main text.

2 Long term intervention

For a long term intervention, the equation for dy/dx (equation (S.1) above) becomes

$$\begin{aligned}\frac{dy}{dx}&=\frac{\beta(t)xy-\gamma y}{-\beta(t)xy}\\&=\begin{cases}\dfrac{1}{R_0x}-1 & t<T_1\\ \dfrac{1}{R_0(1-\phi)x}-1 & T_1\le t\end{cases}\end{aligned}\qquad\text{(S.10)}$$

And thus, by integrating, as for equation (S.2) above,

$$y=\begin{cases}\dfrac{1}{R_0}\ln x-x+c_1 & t<T_1\\ \dfrac{1}{R_0(1-\phi)}\ln x-x+c_2 & T_1\le t\end{cases}\qquad\text{(S.11)}$$

The constants are calculated separately. As in the no intervention case above, $y(0)\approx 0, x(0)\approx 1$, $c_1 = 1$ as in equation (A.4). Then equation (S.4) is used to give the values of $y(T_1)$ and $x(T_1)$ at the point where the intervention starts, in order to calculate c_2. Thus,

$$\begin{aligned}c_2&=y(T_1)-\frac{1}{R_0(1-\phi)}\ln\left[x(T_1)\right]+x(T_1)\\&=\frac{1}{R_0}\ln x(T_1)-x(T_1)+1-\frac{1}{R_0(1-\phi)}\ln\left[x(T_1)\right]+x(T_1)\\&=1-\frac{\phi}{R_0(1-\phi)}\ln x(T_1)\end{aligned}\qquad\text{(S.12)}$$

And so, the dynamics are given by

$$y=\begin{cases}1-x+\dfrac{1}{R_0}\ln x & t<T_1\\ 1-x+\dfrac{1}{R_0(1-\phi)}\ln(x)-\dfrac{\phi}{R_0(1-\phi)}\ln\left(x(T_1)\right) & T_1<t\end{cases}\qquad\text{(S.13)}$$

2.1 Epidemic size

When there is a long term intervention in place the final epidemic size, as $t \to \infty$ and $y(\infty) \to 0$, is given by $a_{LI} = 1 - x(\infty)$, and therefore we can use equation (S.13) to derive:

$$\begin{aligned} 0 &= a_{LI} + \frac{1}{R_0(1-\phi)}\ln(1-a_{LI}) + \frac{\phi}{R_0(1-\phi)}\ln(x(T_1)) \\ \ln(1-a_{LI}) &= -a_{LI}R_0(1-\phi) + \phi\ln(x(T_1)) \\ a_{LI} &= 1 - e^{-a_{LI}R_0(1-\phi)}x(T_1)^{\phi} \\ a_{LI} &= 1 - (1-I(T_1))^{\phi}e^{-a_{LI}R_0(1-\phi)} \end{aligned} \tag{S.14}$$

which is equation (4) in the main text. The approximation to cumulative incidence from equation (S.9) can be used to give an approximate value of the epidemic size for interventions which start during the exponential phase of the unconstrained epidemic.

2.2 Peak prevalence

When there is a long term intervention in place, a localized peak in prevalence can occur before or after the start of the intervention. As derived above, peak prevalence without an intervention in place is given by equation (S.7), and occurs when $x = 1/R_0$. If the intervention is initiated early, then we can perform a similar calculation for peak prevalence during the intervention using the second part of equation (S.10) when $dy/dx = 0$,

$$\begin{aligned} 0 &= \frac{1}{R_0(1-\phi)x} - 1 \\ x &= \frac{1}{R_0(1-\phi)} \end{aligned} \tag{S.15}$$

And the value of peak prevalence during the intervention is calculated using equation (S.13)

$$\begin{aligned} y_{\max_{during}} &= 1 - \frac{1}{R_0(1-\phi)} + \frac{1}{R_0(1-\phi)}\ln\left(\frac{1}{R_0(1-\phi)}\right) - \frac{\phi}{R_0(1-\phi)}\ln(x(T_1)) \\ &= 1 - \frac{1}{R_0(1-\phi)}\left(1 + \ln(R_0(1-\phi)) + \phi\ln(1-I(T_1))\right) \end{aligned} \tag{S.16}$$

as in the main text. Again, the approximation to cumulative incidence prior to the epidemic can be used for early interventions (equation (S.9)).

The next step is to derive conditions under which a peak in prevalence is observed before or during the intervention, or at the time that intervention starts. There are a limited number of scenarios which can occur:

- If the intervention is started after peak prevalence (i.e. $x(T_1) < 1/R_0$), then during the intervention $x < 1/\left(R_0(1-\phi)\right)$ and $dy/dx < 0$ because there are too few susceptibles (equation (S.10)). In this case, the only peak is before intervention, $y_{\max_{NI}}$ (equation A.7).
- If the intervention is started early enough and $x(T_1) > 1/\left(R_0(1-\phi)\right)$, then $dy/dx > 0$ when the intervention is initiated. In this case there will be a peak in prevalence during the intervention, $y_{\max_{during}}$ (equation (A.16)). This may or not may be the global maximum, depending on whether $y(T_1) > y_{\max_{during}}$ or vice versa.
- In addition, there is an intermediate scenario, where the pre-intervention prevalence is still increasing when the intervention is initiated ($x(T_1) > 1/R_0$), but there are too few susceptibles for prevalence to increase when the intervention is initiated ($x(T_1) < 1/\left(R_0(1-\phi)\right)$). In this case prevalence will decrease when the intervention is initiated and peak prevalence will be $y(T_1)$. The window of opportunity for this scenario is largest when ϕ is largest.

3 Short term intervention

For a short term intervention, the equation for dy/dx (equation (S.1) above) becomes

$$\frac{dy}{dx} = \frac{\beta(t)xy - \gamma y}{-\beta(t)xy} = \frac{\gamma}{\beta(t)x} - 1$$
$$= \begin{cases} \dfrac{1}{R_0 x} - 1 & t < T_1 \\ \dfrac{1}{R_0(1-\phi)x} - 1 & T_1 \le t \le T_2 \\ \dfrac{1}{R_0 x} - 1 & t > T_2 \end{cases} \tag{S.17}$$

And thus, by integrating, as for equation (S.2) above,

$$y = \begin{cases} \dfrac{1}{R_0}\ln x - x + c_1 & t < T_1 \\ \dfrac{1}{R_0(1-\phi)}\ln x - x + c_2 & T_1 \le t \le T_2 \\ \dfrac{1}{R_0}\ln x - x + c_3 & t > T_2 \end{cases} \tag{S.18}$$

The constants c_1 and c_2 are as in equation (S.13). For the final part of the curve, the 'initial' conditions are determined by the dynamics before and during the intervention, $y(T_2)$ and $x(T_2)$. Thus,

$$\begin{aligned} c_3 &= y(T_2) - \frac{1}{R_0}\ln\left[x(T_2)\right] + x(T_2) \\ &= 1 - x(T_2) + \frac{1}{R_0(1-\phi)}\ln\left(x(T_2)\right) - \frac{\phi}{R_0(1-\phi)}\ln\left(x(T_1)\right) - \frac{1}{R_0}\ln\left[x(T_2)\right] + x(T_2) \\ &= 1 - \frac{\phi}{R_0(1-\phi)}\ln\left[\frac{x(T_2)}{x(T_1)}\right] \end{aligned} \quad \text{(S.19)}$$

And so, the dynamics are given by

$$y = \begin{cases} 1 - x + \dfrac{1}{R_0}\ln x & t < T_1 \\ 1 - x + \dfrac{1}{R_0(1-\phi)}\ln(x) - \dfrac{\phi}{R_0(1-\phi)}\ln\left(x(T_1)\right) & T_1 \le t \le T_2 \\ 1 - x + \dfrac{1}{R_0}\ln(x) - \dfrac{\phi}{R_0(1-\phi)}\ln\left(\dfrac{x(T_1)}{x(T_2)}\right) & t > T_2 \end{cases} \quad \text{(S.20)}$$

3.1 Epidemic size

When there is a long term intervention in place the final epidemic size, as $t \to \infty$ and $y(\infty) \to 0$, is given by $a_{SI} = 1 - x(\infty)$, and therefore we can use equation (S.20) to derive:

$$\begin{aligned} 0 &= a_{SI} + \frac{1}{R_0}\ln(1-a_{SI}) - \frac{\phi}{R_0(1-\phi)}\ln\left(\frac{x(T_2)}{x(T_1)}\right) \\ \ln(1-a_{SI}) &= -a_{SI}R_0 + \frac{\phi}{(1-\phi)}\ln\left(\frac{x(T_2)}{x(T_1)}\right) \\ a_{SI} &= 1 - \left(\frac{x(T_1)}{x(T_2)}\right)^{\frac{\phi}{1-\phi}} e^{-a_{SI}R_0} \\ a_{SI} &= 1 - \left(\frac{1-I(T_1)}{1-I(T_1+D)}\right)^{\frac{\phi}{1-\phi}} e^{-R_0 a_{SI}} \end{aligned} \quad \text{(S.21)}$$

which is equation (8) in the main text.

3.2 Peak prevalence

When there is a short term intervention in place, a local peak in prevalence can occur before, during or after the intervention. As derived above, peak prevalence without an intervention in place is given by equation (S.7), and occurs when $x = 1/R_0$. Peak prevalence during the intervention is given by equation (A.16), and occurs when $x = 1/\left(R_0\left(1-\phi\right)\right)$. Peak prevalence can also occur after the intervention, if there are enough susceptibles. Peak prevalence will occur when $x = 1/R_0$ (since dy/dx is as before the intervention), and its value will be (from equation (S.20)).

$$\begin{aligned} y_{\max_{post}} &= 1 - \frac{1}{R_0} + \frac{1}{R_0}\ln\left(\frac{1}{R_0}\right) - \frac{\phi}{R_0\left(1-\phi\right)}\ln\left(\frac{x\left(T_1\right)}{x\left(T_2\right)}\right) \\ &= y_{\max_{NI}} - \frac{\phi}{R_0\left(1-\phi\right)}\ln\left(\frac{1-I\left(T_1\right)}{1-I\left(T_1+D\right)}\right) \end{aligned} \tag{S.22}$$

Given general conditions under which peak prevalence can occur before, during or after the intervention, the next step is to derive conditions under which each of these peaks occur. These conditions, expressed in terms of the susceptible pool at the time that an intervention is initiated and lifted, are given in Table 1. Table A1 below gives illustrations of each scenario.

Two additional calculations are required to balance which of pairs of two local peaks in prevalence are highest.

When there is a peak in prevalence after the intervention, it may or not be higher than prevalence when the intervention was initiated, depending on the susceptible pool at the time when the intervention is lifted:

$$\begin{gathered} y\left(T_1\right) > y_{\max_{post}} \\ 1 - x\left(T_1\right) + \frac{1}{R_0}\ln\left(x\left(T_1\right)\right) > 1 - \frac{1}{R_0}\left(1+\ln R_0\right) - \frac{\phi}{R_0\left(1-\phi\right)}\ln\left(\frac{x\left(T_1\right)}{x\left(T_2\right)}\right) \\ x\left(T_2\right) < x_c = x\left(T_1\right)^{1/\phi}\left(R_0 e^{1-R_0 x\left(T_1\right)}\right)^{\frac{1-\phi}{\phi}} \end{gathered} \tag{S.23}$$

When there are peaks during and after the intervention, their relative height also depends on the susceptible pool when the intervention is lifted:

$$y_{\max_{during}} > y_{\max_{post}}$$

$$1-\frac{1}{R_0\left(1-\phi\right)}\left(1+\ln\left(R_0\left(1-\phi\right)\right)\right)-\frac{\phi}{R_0\left(1-\phi\right)}\ln\left(x\left(T_1\right)\right)$$

$$>1-\frac{1}{R_0}\left(1+\ln R_0\right)-\frac{\phi}{R_0\left(1-\phi\right)}\ln\left(\frac{x\left(T_1\right)}{x\left(T_2\right)}\right) \quad \text{(S.24)}$$

$$x\left(T_2\right)<\frac{e^{-1}}{R_0\left(1-\phi\right)^{1/\phi}}$$

And so, the range of outcomes are given by Table 1, A1.

Table A1 Maximum prevalence in the presence of an intervention. Note that $T_1 + D = T_2$ $x(t) = 1 - I(t)$ and $x_c = x(T_1)^{1/\phi}\left(R_0 e^{-(R_0 x(T_1)-1)}\right)^{\frac{1}{\phi}-1}$. As Table 1 in main text, with additional figures.

	Condition	Maximum prevalence	Local peak prior	Increasing prevalence during	Local peak during	Local peak post	Prevalence (No intervention; Prevalence; Start intervention; End intervention)	Susceptibles (Susceptibles; Start intervention; End intervention; 1/R0; 1/(R0(1-phi)); Xc)
Late interventions	$x(T_1+D) < x(T_1) < \frac{1}{R_0}$	$y_{\max_{NI}}$	Y	N	N	N	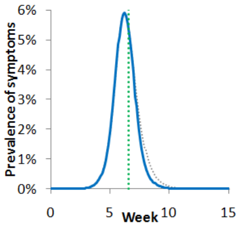	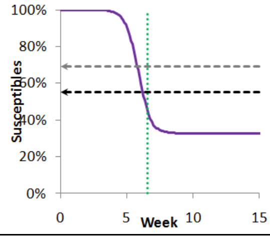
	$x(T_1+D) < \frac{1}{R_0} < x(T_1) < \frac{1}{R_0(1-\phi)}$	$y(T_1)$	N	N	N	N	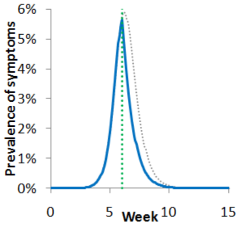	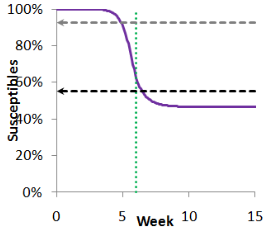

Condition	Maximum prevalence	Local peak prior	Increasing prevalence during	Local peak during	Local peak post	Prevalence<br>No intervention; Start intervention; Prevalence; End intervention	Susceptibles<br>Susceptibles; 1/R0; Start intervention; 1/(R0(1-phi)); End intervention; Xc
$\frac{1}{R_0} < x(T_1+D) < x_c < x(T_1) < \frac{1}{R_0(1-\phi)}$	$y(T_1)$	N	N	N	Y	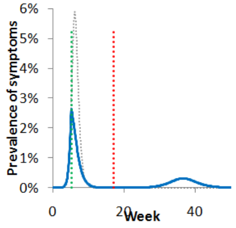	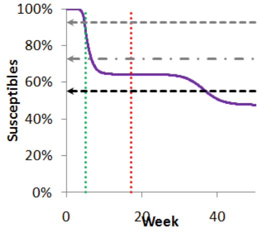
$\frac{1}{R_0} < x_c < x(T_1+D) < x(T_1) < \frac{1}{R_0(1-\phi)}$	$y_{\max_{post}}$	N	N	N	Y	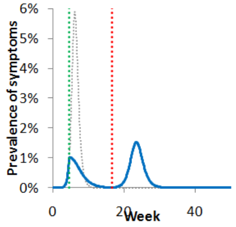	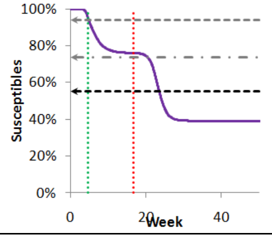
$x(T_1+D) < \frac{1}{R_0} < \frac{1}{R_0(1-\phi)} < x(T_1)$	$y_{\max_{during}}$	N	Y	Y	N	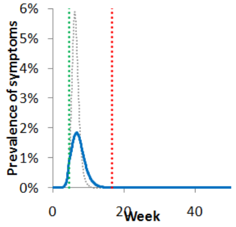	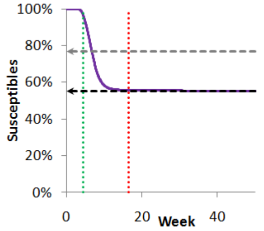

	Condition	Maximum prevalence	Local peak prior	Increasing prevalence during	Local peak during	Local peak post	Prevalence	Susceptibles
							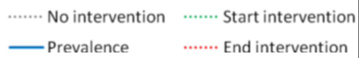	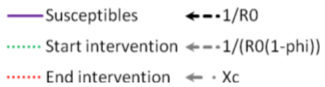
**Early, short interventions**	$\frac{1}{R_0} < x(T_1+D) < \frac{e^{-1}}{R_0(1-\phi)^{1/\phi}} < \frac{1}{R_0(1-\phi)} < x(T_1)$	$y_{\max_{during}}$	N	Y	Y	Y	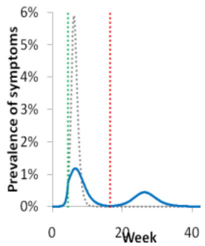	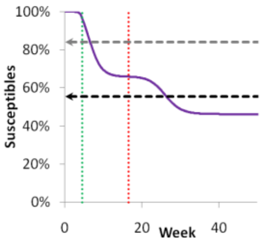
	$\frac{1}{R_0} < \frac{e^{-1}}{R_0(1-\phi)^{1/\phi}} < x(T_1+D) < \frac{1}{R_0(1-\phi)} < x(T_1)$	$y_{\max_{post}}$	N	Y	Y	Y	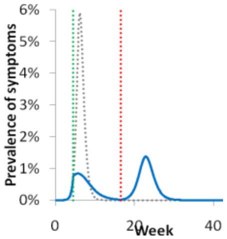	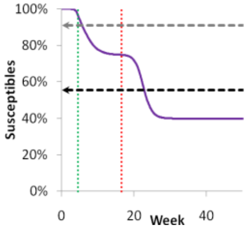
	$\frac{1}{R_0(1-\phi)} < x(T_1+D) < x(T_1)$	$y_{\max_{post}}$	N	Y	N	Y	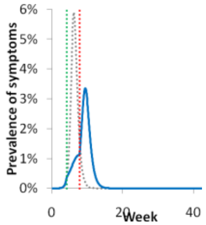	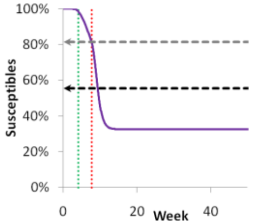
